# A scoping study of component-specific toxicity of mercury in urban road dusts from three international locations

**DOI:** 10.1007/s10653-019-00351-1

**Published:** 2019-06-18

**Authors:** Andrew D. Brown, Bongani Yalala, Ewa Cukrowska, Ricardo H. M. Godoi, Sanja Potgieter-Vermaak

**Affiliations:** 1 School of Science and the Environment, Manchester Metropolitan University, Manchester, UK; 2 AECOM, Regan Way, Nottingham, UK; 3 Molecular Science Institute, University of the Witwatersrand, Johannesburg, South Africa; 4 Environmental Engineering Department, Federal University of Parana, Curitiba, Parana Brazil

**Keywords:** Mercury pollution, Road dust, Pulmonary bioaccessibility, Individual particle analysis

## Abstract

This scoping study presents an investigation of the total and bioaccessible mercury concentrations in road dust (RD) from three international urban sites, where a one-off sampling campaign was conducted at each. This was done to address the hypothesis that the matrix in which mercury is found influences its ability to become accessible to the body once inhaled. For that purpose, the samples were analysed for total and pulmonary bioaccessible mercury and the data compared to the chemical structure of individual particles by SEM. The results obtained from this study suggest that a high mercury content does not necessarily equate to high bioaccessibility, a phenomenon which could be ascribed to the chemical character of the individual particles. It was found that the Manchester samples contained more pulmonary soluble mercury species (as determined by elemental associations of Hg and Cl) in comparison to the other two samples, Curitiba, Brazil, and Johannesburg, South Africa. This finding ultimately underlines the necessity to conduct a site-specific in-depth analysis of RD, to determine the concentration, chemical structure and molecular speciation of the materials within the complex matrix of RD. Therefore, rather than simply assuming that higher bulk concentrations equate to more significant potential human health concerns, the leaching potential of the metal/element in its specific form (for example as a mineral) should be ascertained. The importance of individual particle behaviour in the determination of human health risk is therefore highlighted.

**Electronic supplementary material:**

The online version of this article (10.1007/s10653-019-00351-1) contains supplementary material, which is available to authorized users.

## Introduction

Mercury pollution is of particular interest because of its documented effects on human health and particularly children in utero (Maramba et al. 2010). Neurological and behavioural disorders are commonly observed as a result of mercury inhalation (Hsi et al. 2014; Sondreal et al. 2004). Geological studies indicate that the naturally occurring crustal abundance of Hg is 20–60 µg kg^−1^ (Kabata-Pendias and Mukherjee 2007). However, there is a significant anthropogenic input worldwide, with major sources including fossil fuel combustion, metal production and mining, cement production and various other industries (Mahub et al. 2017). Mercury in these anthropogenically contaminated sites is mostly present as elemental mercury vapour with studies showing a range of 75–99% abundance, with the remainder being present as methyl mercury and the less abundant inorganic mercury (WHO 1990; European Commission 2001). Accordingly, the Environment Agency (UK) has set soil guideline values of 1.0 mg kg^−1^ for elemental mercury in residential soils (Environment Agency 2009). There is, however, evidence to suggest a more realistic guideline for chronic toxicity is 130 µg kg^−1^ (Tipping et al. 2010).

Because of these publicised effects, many studies quantify total mercury in environmental particulate matter (PM), which has the ability to become inhaled, ingested or absorbed through dermal contact. Road dust (RD) is widely considered to be a significant contributor to urban PM, and studies have shown that RD can contribute up to 74% of total suspended particles (Hien et al. 1999; Harrison et al. 1997) and has also been observed to be the largest and second largest contributor to PM_10_ and PM_2.5_, respectively (Landis et al. 2017). Because of the large abundance of RD in urban settings relative to PM, there is a large body of research on Hg concentrations in RD worldwide. Studies in China have reported concentrations of Hg in RD as high as 600 µg kg^−1^ in Xi’an, using cold vapour atomic absorption spectroscopy (CVAAS) (Han et al. 2011). Additionally, publications show reported concentrations of 120 µg kg^−1^ in Nanjing, using ICP-MS (Hu et al. 2011), while Huang et al. (2016) report 220 µg kg^−1^ in Guangzhou using ICP-MS. There have also been several published papers quantifying Hg in other parts of the world. Londonio et al. (2012) found Hg concentrations as high as 1.06 mg kg^−1^ in Buenos Aires, Argentina, using CVAAS. In Matsumoto, Japan, concentrations of Hg in road dust have been observed as high as 26 µg kg^−1^ (Ozaki et al. 2004), and Rodrigues et al. (2014) reported 2.2 mg kg^−1^ in southern Portugal using thermal decomposition atomic absorption spectroscopy in conjunction with a gold matrix modifier.

Based on the results of these studies, inferences have been made on the possible detrimental effects to human health. The ability of mercury to become accessible to the human body, however, is seldom considered. It is evident from the literature cited previously that mercury levels in RD are site specific. It is hypothesised that not only is bulk concentration site specific, but that the bioaccessibility is as well, and the leaching profile onto artificial body fluids is not necessarily correlated with the bulk concentration. To test this hypothesis, RD was collected from three different international urban sites, Manchester (UK), Curitiba (Brazil) and Johannesburg (South Africa). Populations for these cities are believed to be 0.5 million, 1.7 million and 1 million, respectively. These three sites were selected for their diversity of pollutant sources. Mercury pollution in Johannesburg is well documented because of its notoriety as a by-product of the gold mining industry (Lusilao-Makiese et al. 2013; Gichuki and Mason 2014). Bleach production and the textile industry in the 1960s in Manchester have been linked with residual mercury pollution (Vane et al. 2009; Nguetseng et al. 2015). The river Mersey in Manchester still has high mercury concentrations present in water and river sediments (Harland et al. 2000; Nguetseng et al. 2015). Mercury pollution in Curitiba is most likely to originate from the coal-burning power stations of southern Brazil (Ancora et al. 2015).

Representative aliquots of each RD were acid digested, and Hg concentrations were analysed using CVAAS. Simultaneously, aliquots of each RD sample were exposed to artificial lysosomal fluid (ALF), where the resultant leachates were also analysed using CVAAS. General uncertainty among the scientific community is reflected with a wide range of time in which environmental particles are exposed to simulated lung fluids for studies of this nature, for example, 4–24 h (Charrier et al. 2014), 2–630 h (Wragg and Klinck 2007), 1 h–30 days (Colombo et al. 2008), 15 min–72 h (Cabouche et al. 2011) and 1 h–28 days (Potgieter-Vermaak et al. 2012a). This uncertainty is well founded as clinical studies reveal that residence time is affected by an individual’s health (Sturm 2013). In consideration of these studies, a residence time of 24 h was assumed.

Execution of this methodology allows for bioaccessibility via the inhalation route to be elucidated. It was deemed appropriate to only study the inhalation route because it is believed that this is the most appropriate exposure route (Environment Agency 2009). As bioaccessibility of metals is thought to be dependent on the form in which these metals are present within a matrix, an investigation into speciation of mercury-containing compounds within individual particles present in road dust was undertaken using a combined approach of Raman spectroscopy and computer-controlled scanning electron microscope with energy-dispersive X-ray microanalysis (CCSEM–EDX).

## Experimental methodology

### Sampling protocol

Sampling took place at three urban sites, namely Manchester, Curitiba and Johannesburg. Each site was selected for being subject to a high volume of motor vehicles and pedestrians.

At each site, approximately 10 kg of RD was collected from the sampling site in each campaign and air-dried at room temperature (21 °C ± 3 °C). This mass of sample was collected as the previous unpublished work has indicated that 10 kg of wet RD can yield as little as 10 g of < 38 µm RD. Samples were placed in clean brown paper bags on a clean bench, topped with Benchkote^®^ polyethylene-laminated Whatman^®^ paper and left to dry. An aliquot of each RD sample, representative of the rest of the RD sample, was sorted into a smaller brown paper bag. This was weighed daily until the mass stabilised, indicating that the aliquot, and hence the rest of the sample, was dry. Typically, this process took up to 3 weeks depending on relative moisture of RD samples. The resulting dry sediment was separated into different grain size fractions using standard sieve methods. Stainless steel woven wire mesh sieves (200 mm diameter; 50 mm depth: to ISO 3310-1:2000; BS 410-1:2000) were used. The three grain size fractions analysed in this study, < 38 µm, 63–38 µm and 125–63 µm, were attained using sieves of mesh sizes 0.125 mm (US std 120), 0.063 mm (US std 240) and 0.038 mm (US std 400). All equipment used was thoroughly washed to ensure that no metal contamination occurs between samples. A solution of 2% nitric acid was used to clean all non-metal equipment.

The sieves available in South Africa were different, however; therefore, RD from the Johannesburg site was fractioned into samples < 50 µm and 100–50 µm. Sampling took place in Curitiba during August to represent a winter sampling campaign. Similarly, the sampling campaign in Johannesburg occurred during austral winter. The sample from Manchester was collected in November to represent a winter sample.

### Acid digestion of samples

Samples were analysed in triplicate for total mercury using an aqua regia and hydrofluoric acid method described in United States Environmental Protection Agency method 3052 (USEPA 1996). The method suggests exposure of 0.25 g of sample to reagent-grade HNO_3_, HCl and HF in the ratio of 9:3:1 (*v*/*v*/*v*) in PTFE vessels. The mixture was then heated using a PerkinElmer Multiwave 3000 microwave at 800 W for 30 min, followed by 600 W for 20 min. Samples were then allowed to cool at room temperature before the addition of 5% HBO_3_ (6 mL per 1 mL of HF) to precipitate excess fluoride, preventing secondary reactions. Samples were then syringe-filtered before analysis using CVAAS. In addition to the samples, a highly enriched mercury-containing certified reference material (CRM) (ERMCC580-Estuarine sediment) was also digested in triplicate and analysed in the same manner.

### Artificial lysosomal fluid dissolution

Artificial lysosomal fluid was prepared according to Colombo et al. (2008), where reagents listed in Table 1 were added sequentially to deionised water, and each reagent was dissolved before the next was added. The experimental protocol started with 0.15 g ± 0.0015 g of each RD sample being weighed out into a centrifuge vial (in triplicate), 15 mL of ALF was added to each vial, sealed and shaken by hand for 10 s, and each vial was then placed in a shaking incubator for 24 h. On removal from the shaking incubator, samples were allowed to cool to room temperature for 10 min before being filtered using 0.2-μm Whatman^®^ PVDF syringe filters into clean universal vials. The samples were then acidified using spectral grade HNO_3_ (310 µL), to achieve a 2% *w*/*v* acid solution and stored at 4 °C until analysis could be performed.

**Table 1:** Artificial lysosomal fluid reagent list

Reagent	ALF (Colombo et al. 2008) (g/L)
Sodium chloride	3.21
Magnesium chloride	0.05
Disodium hydrogen phosphate	0.071
Sodium sulphate	0.039
Calcium chloride dihydrate	0.128
Sodium citrate dihydrate	0.077
Glycine	0.059
Citric acid	20.8
Sodium hydroxide	6
Sodium tartrate	0.09
Sodium lactate	0.085
Sodium pyruvate	0.086
Final pH	4.5

### Cold vapour atomic absorption spectroscopy

A PerkinElmer FIMS 400 Flow Injection Mercury System with a PerkinElmer AAnalyst 200 atomic absorption spectrometer and AS-91 autosampler was used to quantify mercury. The process injects the 500 µL of sample into a reaction vessel, along with 1.1% (*m*/*v*) SnCl_2_ in 3% (*v*/*v*) HCl as a reducing agent, to ensure all Hg is in the 0 oxidation state (Harris-Hellal et al. 2009). The reaction vessel is then purged with Ar to liberate all gaseous Hg. The mercury vapour and Ar carrier gas continue through the system to a 15-cm quartz tube with a hollow cathode lamp (190 mA lamp current, 0.7 nm slit width, 253.7 nm wavelength of radiation) at one end and a solar-blind detector at the other. Concentration of samples is determined relative to standards. In each set of analysis, matrix-matched standards of 0, 1, 2, 5 and 10 µg L^−1^ were used, and both times regression line > 0.99 was achieved. To ensure no drift in instrument detection took place, quality control standards of 0.5 and 1 µg L^−1^ were analysed every ten samples, and recovery was consistent, between 94% and 99% in all cases. In addition to this, the CRM was analysed at the end of each analysis, giving a mean recovery for the CRM replicates of 99.0%, with RSD (%) of 3.70. Limit of detection for this instrumentation was found to be 0.0152 µg L^−1^ based on the seven blanks of ALF and 0.0095 µg L^−1^ based on seven blanks of the acid digestion solution.

### Mercury clustering

Raman spectroscopy can be used for gaining a comprehensive assessment of individual particles (Taylor and Robertson 2009; Potgieter-Vermaak et al. 2012a). These studies, concerned with characterising individual particles, show that there is scope for analysing particles within the interest of health. Although the process can provide accurate structural information of particles of both amorphous and crystalline nature, as well as information on the heterogeneous character of an individual particle, it cannot provide reliable quantitative information on the abundance of these compounds due to a limited volume of sample analysed. Preceding the Raman analyses with a semi-quantitative method, computer-controlled scanning electron microscope with energy-dispersive X-ray microanalysis (CCSEM–EDX) has the potential to provide a more quantitative approach. In addition, it provides associations of elements in a particle which would be used intuitively with the Raman analysis to confirm the chemical structure of the compound.

Kandler et al. (2011) have shown that particles can be clustered based on atomic ratios, determined by CCSEM–EDX, to obtain a class of particle (silicates, calcites, etc.). However, the analysis fails to inform us how these elements are present. Anaf et al. (2012) showed that clustering can be done based on atomic ratios. For example, consider the chemical formula for dolomite, CaMg(CO_3_)_2_, where the atomic ratio of Ca/Mg is 1:1. This then informs one of the criteria within the clustering criteria for dolomite cited by Anaf et al. (2012), which is 0.33 < Mg/Ca < 3, and Mg and Ca denote the moles for each element, calculated from the elemental concentration obtained by EDX for that specific particle. This represents a large margin of error for the atomic ratio; however, the other criterion used to define a dolomite particle is Mg + Ca/AE > 0.45, where AE represents the sum of moles of every other element quantified. This criterion is used to ensure that the main constituents of the particle are Mg and Ca; therefore, with the albeit relaxed atomic ratio there is a large degree of certainty that this particle is dolomite.

As the aim of this study is to better understand the microchemical structure of individual particles containing mercury to understand how it influences the toxicity, the methods used by Kandler et al. (2011) and Anaf et al. (2012) are adapted for use with mercury compounds. This study assumes that a large amount of mercury-containing compounds are present as inclusions on larger particles, rather than existing as individual mercury-rich particles. Therefore, it may be necessary to dispense off clustering criteria which characterises a compound as the main constituent of a particle and place more emphasis on the stoichiometric ratios between elements of known mercury compounds but in a stricter manner, to ensure their presence.

#### Raman spectroscopy

A Thermo Scientific DXR Raman microscope equipped with a 532-nm diode-pumped solid-state laser and 50× objective was used. The instrument features a Thermo Scientific calibration tool which, when placed on the microscope stage, allows the user to perform a simultaneous alignment and calibration by placing the cross-hairs within the eyepieces on a pinhole. The surface beneath the pinhole is able to move, switching between copper and polystyrene, to align and calibrate the laser, respectively. The alignment and calibration feature of the instrument is user-initiated and then controlled by the instrument to ensure correct execution. Samples for Raman spectroscopy analysis were prepared on 12.5-mm plastic sticky tabs adhered to a glass slide. A small number of particles were tipped onto a laminated piece of paper, shaken gently to distribute them over a small area so that particles are adequately separated and then mounted on a sticky tab on a glass slide. The user then manually analyses particles in a serpentine pattern, starting in the top-left corner, rastering along to the right, then moving down and rastering along to the left. The benefit of carrying out this process on a glass slide is that it enables the user to observe coordinates on the slide, allowing the user to carry out analysis over several sessions if necessary. Two hundred particles per sample were analysed, and this quantity is deemed adequate to be representative of a sample. In the literature, analysis of 50 particles is generally deemed representative (Potgieter-Vermaak et al. 2012b; Jentzsch et al. 2013).

Analysis of resulting spectra was carried out using the inbuilt Thermo Scientific Spectral Database as a first estimate. Further verification of spectra was carried out using CrystalSleuth computer program, which uses the RRUFF database (RRUFF.info). CrystalSleuth has been well used as a method for elucidating the composition of minerals (Lammers et al. 2012; Wang et al. 2017; Kereszturi et al. 2017).

#### Computer-controlled scanning electron microscopy with energy-dispersive X-ray microanalysis

Computer-controlled EDX analysis, using a Zeiss Supra 40VP field emission CCSEM–EDX, was used to obtain individual particle size and chemical composition. Samples were mounted by dispersion onto silver foil, and a backscattered electron detector was used at 1000× magnification to acquire an image of the stub surface. EDAX Genesis software was used to control the instrument, generating a series of images spiralling out from the centre of the stub, essentially mapping it. The program recognises particles based on their dark colour relative to the background, where greyscale sensitivity is programmed by the operator beforehand. The program measures the *x* and *y* ferets of a particle based on the number of pixels. Each particle was exposed to an acquisition of 15 s at 25 keV giving metal concentrations as percentage of each particle based on signal peaks at discrete KeV values. Elemental data were then separated into clusters based on mole fractions per element, as described by Kandler et al. (2011).

## Results

### Total mercury concentrations

**Fig. 1:**
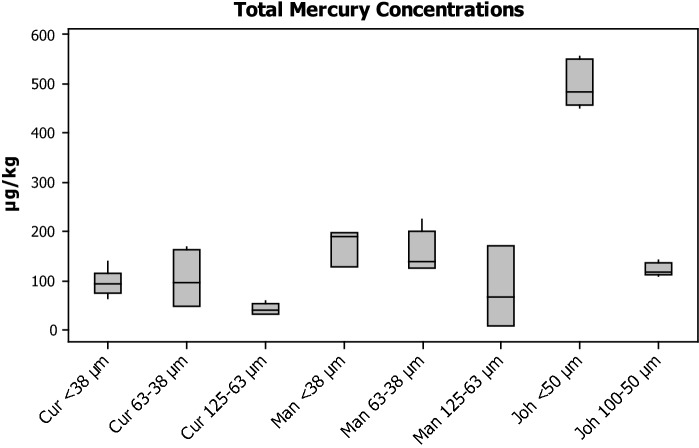
Box plots denoting the median acid soluble concentrations of Hg as determined by CVAAS for each sampling location and size fraction (where *Cur* Curitiba, *Man* Manchester and *Joh* Johannesburg)

Bulk mercury concentrations are displayed in Fig. 1 as median values with interquartile range and in Table 2 with median and %RSD. Mercury concentrations for the Curitiba site showed comparable concentrations in the < 38 µm and 63–38 µm fractions, with median values of 94 µg kg^−1^ and 95 µg kg^−1^, respectively. The 125–63 µm fraction was significantly less enriched, with a concentration of 41 µg kg^−1^. A similar pattern was observed with the Manchester samples, where the < 38 µm and 63–38 µm fractions were similar, with medians of 190 µg kg^−1^ and 139 µg kg^−1^, respectively; again the larger fraction was significantly less enriched with a median concentration of 67 µg kg^−1^. The Johannesburg sample showed significantly higher concentrations in the finer fraction relative to the coarser fraction, with concentrations of 482 µg kg^−1^ and 117 µg kg^−1^, respectively. This large difference between the fractions is not observed in the Curitiba or Manchester samples, which implies that the Hg source or RD matrix has an influence on Hg distribution in size fractions of RD. The pattern observed in each of these samples, whereby the concentration of mercury decreases with increasing grain size, is reported by Brown et al. (2015) with reference to trace metals in RD.

**Table 2:** Acid soluble concentrations of Hg as determined by CVAAS for each sampling location and size fraction (where *Cur* Curitiba, *Man* Manchester and *Joh* Johannesburg)

	Cur < 38 µm	Cur 63–38 µm	Cur 125–63 µm	Man < 38 µm	Man 63–38 µm	Man 125–63 µm	Joh < 50 µm	Joh 100–50 µm
Concentration (µg kg^−1^)	94.0	95.4	40.6	189.8	139.5	68.0	482.3	117.4
%RSD	24.6	49.9	22.3	16.8	27.7	102.6	8.6	10.3

Crustal abundance of mercury is believed to be in the range of 20–60 µg kg^−1^ (Kabata-Pendias and Mukherjee 2007); however, reported concentrations in soil vary significantly on a global scale due to widespread pollution over centuries. In this manner, it is difficult to determine an appropriate background concentration for any given location. Reported “baseline” concentrations of mercury in soil from a site in South Africa are 0.5–28.8 µg kg^−1^ (Walters et al. 2017). It is clear that the Johannesburg dust samples were well above the background value, and it would be fair to say that anthropogenic enrichment has occurred. The sampling site was located close to gold mine tailings storage facilities, and due to the prevailing wind direction, it would evidently be enriched with Hg, which was used in the gold extraction process. Average mercury concentrations in Brazilian soils have been found in the order of 77 µg kg^−1^ (Barbo et al. 2003). The Hg concentrations in both fractions have increased by 23% and could possibly be ascribed to nearby cement industries. Both of these studies indicate a fair agreement in terms of existing concentrations of mercury, where the studies indicate a concentration without anthropogenic influence, however feasible that is. Ander et al. (2013) report “background” concentration of mercury as 1900 µg kg^−1^ in urban areas of the UK. It should be noted that this study indicates that this value includes anthropogenic input. This highlights the difficulty comparing data as presented in this study with background/baseline values, when these terms are used interchangeably and, in many incidences, incorrectly. Tipping et al. (2011) determined a soil critical limit of 130 µg kg^−1^, above which soil biota will be damaged. Using that as a guideline value, it is also evident that the < 38 µm fraction exceeded this value and provides some evidence that the UK RD has been impacted by anthropogenic sources of Hg. Tipping et al. used a large historical database of Hg soil data, but also conducted a sampling campaign in the north-west of England. The mean concentration level found in those samples was 389 µg kg^−1^ which is nearly twice higher than what our study reports. Unfortunately, no information on the location of the samples was given.

Table 3 summarises published concentrations of mercury in RD and the fraction size analysed, many of which were conducted in China and none could be found for UK road dust or specifically in Manchester. The concentrations found in the samples in this study were generally in the same range, perhaps slightly less concentrated than those found worldwide. It is important to note, however, that many of these studies only included one size fraction which was generally larger than those from this study. This could potentially indicate that finer fractions at each of these sites could be significantly more enriched than reported if enrichment increases with decreasing size fraction, as demonstrated by this study. There are two studies cited below (Londonio et al. 2012; Wang et al. 2017) that considered size-fractioned dust, reported on RD collected from different urban sites in Buenos Aires. Londonio et al. (2012) reported an average concentration of 1480 µg kg^−1^ (ranging from < LoD to 3510 µg kg^−1^) for the size fraction < 37 µm and 1640 µg kg^−1^ (ranging from 490 to 4630 µg kg^−1^) for the size fraction < 55 µm. They also report a significant enrichment of Hg in the finer fractions as we have shown in Table 2, with larger fractions having more than two times lower concentrations. The Londonio et al. (2012) paper elude to the large variation in data across sites, which corroborates with our findings, although these concentrations are much higher than those found in Curitiba (by 17 times for the smallest fraction), Johannesburg (by three times for the smallest fraction) and Manchester (by 8 times for the smallest fraction). Wang et al. (2017) on the other hand report values much closer to the three cities we investigated and also indicated the significant enrichment in the finer fraction.

**Table 3:** Literature-reported Hg concentrations from international sites, along with appropriate grain size fraction

Citation	Hg (µg kg^−1^)	Grain size (µm)	Location
Ozaki et al. (2004)	26	< 2000	Japan
Han et al. (2011)	600	< 1000	China
Hu et al. (2011)	120	< 63	China
Londonio et al. (2012)	1480	< 37	Argentina
Wang et al. (2017)	250	< 63	China
Huang et al. (2016)	220	< 100	China
Rodrigues et al. (2014)	2200	< 250	Portugal

### Micro-Raman data

Raman spectroscopic analysis was carried out as described in “Raman spectroscopy” section. of this paper. No mercury-containing particles were observed in any of the samples. It is possible that the number of particles analysed may have been too small, or that the mercury-containing compounds may not be Raman active. Earlier work on road dust at the location in Manchester obtained spectra for an arzakite particle (Hg_3_S_2_Br_2_) (Potgieter-Vermaak et al. 2012a). In an online search of scientific literature, this is the only mercury species that has been characterised in road dust.

### CCSEM–EDX data

The elemental composition of each particle was determined as described in “Computer-controlled scanning electron microscopy with energy-dispersive X-ray microanalysis” section. The number of mercury-containing particles in the Manchester, Johannesburg and Curitiba samples is displayed in Table 4. Results presented in Table 4 indicate that the number of particles containing Hg is considerable in each sample, ranging from 4.5 to 51.7%. It should be noted that in the majority of these, Hg is only observed in small quantities and is not the dominant chemical element. It is observed from Table 4 that the portion of Hg-containing particles decreases with increasing grain size in the Manchester and Curitiba samples; this is not the case in the Johannesburg samples. This indicates an agreement with the CVAAS analysis for the Manchester and Curitiba samples but not the Johannesburg samples. The abundance of Hg in the Brazilian particles is much higher than the Johannesburg and Manchester particles, and since this does not directly correlate with the total concentrations, it indicates that the Curitiba particles contain proportionally less Hg than the other two areas investigated. This could potentially result in a higher risk due to a larger surface area exposed to body fluids, either through ingestion or inhalation. In addition, it suggests that the Curitiba samples may be from a different form. Further analysis of this is provided in the supplementary section of this paper, where a mercury clustering technique is used in an attempt to provide a more detailed analysis, novel for this application.

**Table 4:** Number of mercury-containing particles within each sample

Sample	Mercury-containing particles	Proportion of all particles (%)
Man < 38 µm	139	14
Man 63–38 µm	61	6.1
Man 125–63 µm	45	4.5
Joh < 50 µm	105	11
Joh 100–50 µm	345	35
Cur < 38 µm	517	52
Cur 63–38 µm	440	44
Cur 125–63 µm	369	37

It was observed that Hg-containing particles were commonly found with other metals synonymous with traffic pollution. The frequency with which the Hg particles were observed with S, Zn, Cu and Pb is displayed in Table 5. Table 5 indicates that there is a large presence of particles containing Hg and S in the Manchester samples relative to the larger size fraction of the Johannesburg sample and all size fractions of the Curitiba samples. Sulphur is an abundant element in environmental samples (Brown et al. 2015) but is also an indicator of traffic-related pollution. The high correlation between Hg and S in the Manchester samples can be contributed to the heavy bus traffic on Oxford Road, therefore mainly from diesel exhaust emissions. The Johannesburg correlation in the small fraction could also be related to diesel emissions as the diesel fuel in South Africa is still above the 50 ppm level in most cases. In Curitiba, however, different fuels are used that are inherently low in sulphur. Zn is also a traffic-related pollutant, and it is observed that there is a near 100% correlation with Hg and Zn at all sites and all size fractions. Cu and Pb, both associated with mechanical wear of vehicles, also show high correlations at all sites and size fractions. Since the Zn could also be associated with mechanical wear on vehicles, for example, tire wear, it could be discerned that the Hg at Curitiba is mainly due to mechanical wear and in both Johannesburg and Manchester there is a contribution from both liquid fuel and mechanical wear.

**Table 5:** Portion of Hg particles observed with each of S, Zn, Cu and Pb

Sample	S (%)	Zn (%)	Cu (%)	Pb (%)
Man < 38 µm	17.3	100.0	100.0	89.2
Man 63–38 µm	93.4	95.1	93.4	55.7
Man 125–63 µm	84.4	84.4	88.9	73.3
Joh < 50 µm	23.8	100.0	100.0	96.2
Joh 100–50 µm	1.7	100.0	100.0	100.0
Cur < 38 µm	0.8	100.0	100.0	100.0
Cur 63–38 µm	0.2	100.0	100.0	100.0
Cur 125–63 µm	0.3	100.0	100.0	99.5

### Artificial lysosomal fluid leaching concentrations of mercury

**Fig. 2:**
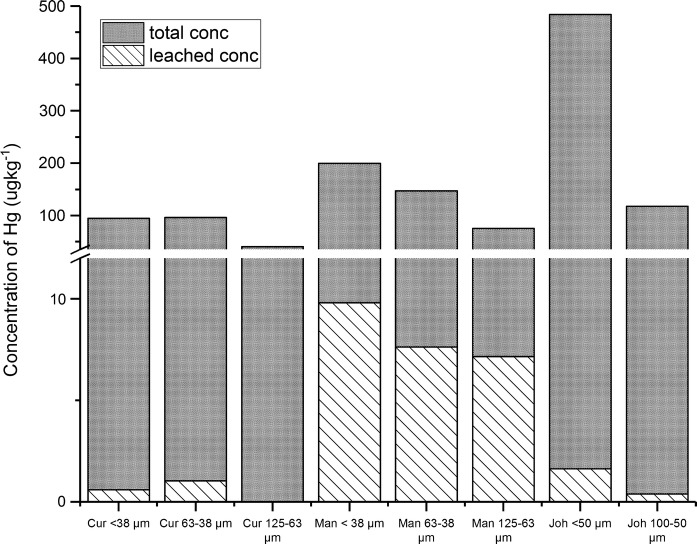
Leached and total mercury concentrations as determined by CVAAS (where *Cur* Curitiba, *Man* Manchester and *Joh* Johannesburg)

Leached and total concentrations of mercury are displayed by site in Fig. 2. This figure contains a break on the *Y*-axis to better present the portion of Hg that leaches. It can be observed from Fig. 2 that the total leaching concentration is significantly higher in the Manchester samples than that of the other two sites. It is also observable that the Hg concentration of the leachates is more enriched in the finer fractions for both the Manchester and the Johannesburg samples. This is not the case for the Curitiba samples, where the 63–38 µm fraction shows the most leaching in terms of absolute concentration. The leached concentrations of Hg in the 125–65 µm fraction of the Curitiba sample are missing from the figure, as the concentrations were found to be below the limit of detection for each replicate.

**Fig. 3:**
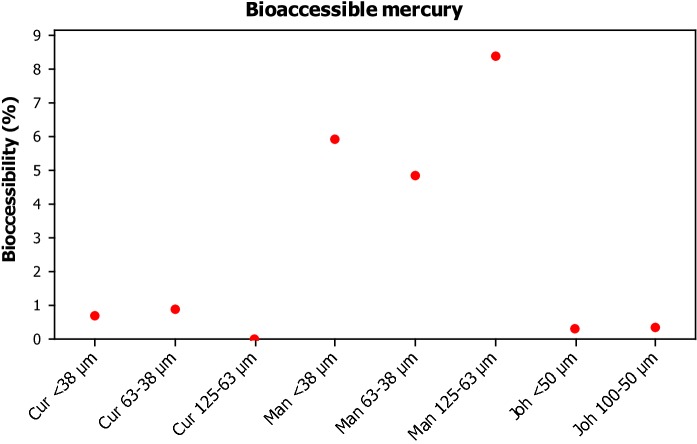
Dot plot denoting the bioaccessible concentrations of Hg for each sampling location and size fraction (where *Cur* Curitiba, *Man* Manchester and *Joh* Johannesburg)

For the remainder of this results section, Hg concentrations will be referred to in the context of bioaccessibility as a percentage. Bioaccessibility is referred to as the concentration of Hg able to leach into ALF over the total concentration in each sample displayed as a percentage. Bioaccessibility of Hg from each sample is displayed in Fig. 3 and Table 6. Table 6 includes standard deviation, which is determined by propagation of errors (Miller and Miller 2010). Leached Hg concentration in the 125–63 µm fraction of the Curitiba site was found to be below the limit of detection for each replicate, and this is signified in Table 6 with “<  LOD”.

**Table 6:** Bioaccessibility of mercury for each sampling location and size fraction (where *Cur* Curitiba, *Man* Manchester and *Joh* Johannesburg)

	Cur < 38 µm	Cur 63–38 µm	Cur 125–63 µm	Man < 38 µm	Man 63–38 µm	Man 125–63 µm	Joh < 50 µm	Joh 100–50 µm
Bioaccessibility (%)	0.70	0.88	< LOD	5.94	4.87	8.40	0.32	0.33
SD	0.64	0.79		0.29	0.26	0.85	1.00	0.95

Bioaccessibility results from this study show an interesting trend whereby the Manchester samples, of which 4.87–8.4% leached, are significantly more bioaccessible than those of Curitiba and Johannesburg, 0–0.88% and 0.32–0.33%. This would suggest that Hg is either present in a different form or differently bonded/absorbed/associated with the matrix, in the Manchester samples. Under the slightly acidic conditions of the ALF, a higher bioaccessibility of Hg indicates that the mercury may be present as the hydroxide or chloride salt. During CCSEM–EDX analysis performed on the samples, as reported in Sect. 3.3, it was found that the mercury was frequently associated with sulphur or copper, rendering it essentially insoluble. The clustering of the Manchester sample on the other hand indicated the presence of chloride salts mainly, which due to its solubility would explain the increased bioaccessibility. The relative insolubility of Hg in the Curitiba and Johannesburg samples could potentially indicate the prevalence of Hg^0^ and Hg organic species (Bloom et al. 2003). In support of this, Malehase et al. (2016) have shown that Hg^0^ prevails in mine tailings in Johannesburg, which can ultimately pollute RD. In the context of toxicity to humans, the literature confirms the toxicity of mercury in the form of organic compounds, inorganic compounds and elemental mercury (Sysalova et al. 2017; Mahub et al. 2017). There is, however, evidence to suggest the effects of organic and elemental mercury are more potent (Lohren et al. 2015).

Referring back to the results of this study, when comparing size fractions within the same sampling location, the < 38 µm and 63–38 µm fraction of RD from Curitiba had similar bioaccessibility (0.70 and 0.88%). Similarly, the two size fractions from the Johannesburg samples were comparable in terms of bioaccessibility (0.32 and 0.33%). This would suggest that in both cases, mercury is found in the same form and that corresponding RD matrix has no influence on this. Bioaccessibility in the 125–63 µm Curitiba samples was found to be below the detection limit, which suggests that either the form in which the mercury is present differs from the two finer fractions from the same site or, most likely, the RD matrix is different within this larger fraction. It is documented that when Hg^2+^ or Hg^0^ is introduced to a soil, the organic and inorganic content of the soils plays a significant role in dictating the form in which Hg is then found (Obrist et al. 2011; Grangeon et al. 2012). It is likely that a similar process occurs within RD and changes in mineral abundance between size fractions can result in this.

Studies in the academic literature on bioaccessibility of Hg via the inhalation route are sparse. There are currently two studies published which explicitly quote concentrations of leachates or bioaccessibility. Both of these studies, however, have different experimental designs to our work, which makes comparisons slightly difficult. Nevertheless, although Huang et al. (2016) published bioaccessibility of Hg in lung serum at 20.66% when the < 2.5 µm portion of RD was leached, which is much more than what we report, this is not a surprising result as these particles will have a much larger surface, facilitating higher leaching concentrations. Rodrigues et al. (2014) report a bioaccessibility figure of 4.1% for Hg from urban soil < 250 µm using Gamble’s solution, which is similar to the values we report for Manchester, albeit in the ALF which has a much lower pH than Gamble’s solution. They have also shown that the bioaccessibility is dependent on the fractionation of Hg in the soil, which corroborates with our investigation where we show with SPA that the chemical speciation of the Hg is different.

## Conclusion

The results of this study conclude that the bulk concentration and bioaccessibility of Hg vary significantly site to site. This has been demonstrated by the substantial observed differences in bulk and bioaccessible concentrations of Hg among the three sites. This is particularly evident while comparing the difference in bioaccessibility between the Manchester site and the other two cities. The study has shown that bioaccessibility is up to an order of magnitude higher in the Manchester samples. The results obtained here would suggest that the source of Hg at each site has a considerable influence ultimately on the bioaccessibility of Hg.

The second interesting conclusion is that the results indicate that the RD matrix present at each site also influences the form in which Hg is present, and this is manifested in this study by differences in bioaccessibility between size fractions at the Curitiba site. It can be seen in the results section of this study that the bioaccessibility of Hg in the two finer fractions from the Curitiba site is 0.70% and 0.88%; however, the larger size fraction leached no measurable quantity of Hg. It has been demonstrated in several studies that the mineral composition of soils has an influence on the Hg species found. This study is the first to show this influence within RD and indeed within different size fractions of RD from a single site.

This study also presents the potential dual approach to chemical clustering within individual particles in the supplementary section. In this incident, there were no substantiated results to report. However, the techniques exhibit the potential for use of Raman spectroscopy with SEM–EDX. The two key conclusions of this study support the necessity to examine metal concentrations on a site-by-site basis because it is the form in which a metal is present, which ultimately dictates its potential harm to human health. This study also presents the importance of examining metals in terms of their mobility in biological fluids, in addition to merely obtaining total concentrations.

## Electronic supplementary material

Below is the link to the electronic supplementary material.

Supplementary material 1 (DOCX 14 kb)

## References

[CR1] Anaf W, Horemans B, Van Grieken R, Da Wael K (2012). Chemical boundary conditions for the classification of aerosol particles using computer controlled electron probe microanalysis. Talanta.

[CR2] Ancora MP, Zhang L, Wang S, Schriefels J, Hao J (2015). Economic analysis of atmospheric mercury emission control for coal-fired power plants on China. Journal of Environmental Sciences.

[CR3] Ander EL, Johnson CC, Cave MR, Palumbo-Roe B, Nathanail CP, Murray Lark R (2013). Methodology for the determination of normal background concentrations of contaminants in English soil. Science of the Total Environment.

[CR4] Bloom NS, Preus E, Katon J, Hiltner M (2003). Selective extractions to assess the biogeochemically relevant fractionation of inorganic mercury in sediments and soils. Analytical Chimica Acta.

[CR5] Barbo ES, Angelica RS, Silva AP, Faial KRF, Mascarenhas AFS, Santos ECO, Jesus IF, Loureiro ECB (2003). Assessment of mercury levels in soils, waters, bottom sediments and fishes of Acre State in Brazilian Amazon. Water Soil and Air Pollution.

[CR6] Brown A, Barrett JE, Robinson H, Potgieter-Vermaak S (2015). Risk assessment of exposure to particulate output of a demolition site. Environmental Geochemistry and Health.

[CR7] Cabouche J, Esperanza P, Bruno M, Alleman LY (2011). Development of an in vitro method to estimate lung bioaccessibility of metals from atmospheric particles. Journal of Environmental Monitoring.

[CR8] Charrier JG, McFall AS, Richards-Henderson NK, Anastasio C (2014). Hydrogen peroxide formation in a surrogate lung fluid by transition metals and quinones present in particulate matter. Environmental Science and Technology.

[CR9] Colombo C, Monhemius AJ, Plant JA (2008). Platinum, palladium and rhodium release from vehicle exhaust catalysts and road dust exposed to simulated lung fluids. Ecotoxicology and Environmental Safety.

[CR10] Environment Agency. (2009). *Soil guideline for mercury in soil*. Science Report SC050021.

[CR11] European Commission. (2001). Ambient air pollution by mercury (Hg) position paper. http://ec.europa.eu/environment/air/pdf/pp_mercury.pdf#. Last accessed April 26, 2018.

[CR12] Gichuki SW, Mason RP (2014). Wet and dry deposition of mercury in Bermuda. Atmospheric Environment.

[CR13] Grangeon S, Geudron S, Asta J, Sarret G, Charlet L (2012). Lichen and soil as indicators of an atmospheric mercury contamination in the vicinity of a chlor-alkali plant (Grenoble, France). Ecological Indicators.

[CR14] Han S, Youn JS, Jung YW (2011). Characterisation of PM10 and PM2.5 source profiles for resuspended road dust collected using mobile sampling methodology. Atmospheric Environment.

[CR15] Harland BJ, Taylor D, Wither A (2000). The distribution of mercury and other trace elements on the sediments of the Mersey Estuary over 25 years 1974–1998. Science of the Total Environment.

[CR51] Harris-Hellal J, Vallaeys T, Garnier-Zarli E, Bousserrhine N (2009). Effects of mercury on soil microbial communitites in tropical soils of French Guyana. Applied Soil Ecology.

[CR16] Harrison RM, Smith DGT, Pio CA, Castro LM (1997). Comparative receptor modelling study of airborne particulate pollutants in Birmingham, Unite Kingdom, Coimbra (Portugal) and Lahore (Pakistan). Atmospheric Environment.

[CR17] Hien PD, Binh NT, Truong Y, Ngo NTN (1999). Temporal variations of source impacts at the receptor, as derived from particulate monitoring data in Ho Chi Minh City, Vietnam. Atmospheric Environment.

[CR18] Hsi HC, Jiang CB, Yang TH, Chien LC (2014). The neurological effects of prenatal and postnatal mercury/methylmercury exposure on 3-year-old children in Taiwan. Chemosphere.

[CR19] Hu X, Zhang Y, Luo J, Wang T, Lian H, Ding Z (2011). Bioaccessibility and health risk of arsenic, mercury and other metals in urban street dusts from a mega-city, Nanjing, China. Environmental Pollution.

[CR20] Huang J, Li F, Zeng G, Liu W, Huang X, Ziao Z, Wu H, Li X, He X, He Y (2016). Integrating hierarchical bioavailability and population distribution into potential eco-risk assessment of heavy metals in road dust: A case study in Xiandao District, Changsha city, China. Science of the Total Environment.

[CR21] Jentzsch PV, Kampe B, Ciobota V, Rosch P, Popp J (2013). Inorganic salts in atmospheric particulate matter: Raman spectroscopy as an analytical tool. Spectrochimica Acta Part A: Molecular and Biomolecular Spectroscopy.

[CR22] Kabata-Pendias A, Mukherjee AB (2007). Trace elements from soil to human.

[CR23] Kandler K, Leike K, Benker N, Emmel C, Kupper M, Muller-Ebert D, Scheuvens D, Schladitz A, Schutz L (2011). Electron microscopy of particles collected at Praia, Cape Verde, during the Saharan Mineral Dust Experiment: Particle chemistry, shape, mixing state and complex refractive index. Tellus.

[CR24] Kereszturi A, Gyollai I, Kereszty Zs, Kiss K, Szabo M, Szalai Z, Ringer M, Veres M (2017). Analyzing raman—infrared spectral correlation in the recently found meteorite Csátalja. Spectrochimica Acta Part A: Molecular and Biomolecular Spectroscopy.

[CR25] Lammers L, Naujoks C, Berr K, Depprich R, Kunler N, Meyer U, Langenbach F, Luttenberg B, Kogler G, Wiseman H, Handschel J (2012). Impact of DAG stimulation on mineral synthesis, mineral structure and osteogenic differentiation of human cord blood stem cells. Stem Cell Research.

[CR26] Landis MS, Pancras JP, Graney JR, White EM, Edgerton ES, Legge A, Percy KE (2017). Source appointment of ambient fine and coarse particulate matter at the fort Mckay community site, in the Athabascca Oil Sands Region, Alberta, Canada. Science of the Total Environment.

[CR27] Lohren H, Blagojevic L, Fitkau R, Ebert F, Schildknecht S, Leist M, Schwerdtle T (2015). Toxicity of organic and inorganic mercury species in differentiated human neurons and human astrocytes. Journal of Trace Elements in Medicine and Biology.

[CR28] Londonio A, Fujiwara F, Rebagliati RJ, Gomez D, Smichowski P (2012). Determination of mercury in size fractionated road dust samples by flow injection-cold-vapour-atomic absorption spectrometer. Microchemical Journal.

[CR29] Lusilao-Makiese JG, Cukrowska EM, Tessier E, Amouroux D, Weiersbye I (2013). The impact of post gold mining on mercury pollution in the West Rand region, Gauteng, South Africa. Journal of Geochemical Exploration.

[CR30] Mahub KR, Krishnan K, Naidu R, Andrews S, Megharaj M (2017). Mercury toxicity to terrestrial biota. Ecological Indicators.

[CR31] Malehase T, Daso AP, Okonkwo JO (2016). Determination of mercury and its fractionation products in samples from legacy use of mercury amalgam in gold processing in Randfontein, South Africa. Emerging Contaminants.

[CR32] Maramba NPC, Reyes JP, Francisco-Rivera AT, Panganiban LCR, Dioquino C, Dando N, Timbang R, Akagi H, Castillo T, Quintoriano C, Afuang M, Matsutama A, Eguchi T, Fuchigami Y (2010). Environmental and human exposure assessment monitoring of communities near an abandoned mercury mine in the Philippines: A toxic legacy. Journal of Environmental Management.

[CR33] Miller JN, Miller JC (2010). Statistics and chemometrics for analytical chemistry.

[CR34] Nguetseng R, Fliender A, Knopj B, Lebreton B, Quack M, Rudel M (2015). Retrospective monitoring of mercury in fish from selected European freshwater and estuary sites. Chemosphere.

[CR35] Obrist D, Johnson DW, Lindberg SE, Luo Y, Hararuk O, Branco R, Battles JJ, Dail DB, Edmonds RL, Monson RK, Ollinger SV, Pallardy SG, Pregitzer KS, Todd DE (2011). Mercury distribution across 14 U.S. Forests. Part I: Spatial patterns of concentrations in biomass, litter, and soils. Environmental Science and Technology.

[CR36] Ozaki H, Watanabe I, Kuno K (2004). As, Sb and Hg distribution and pollution sources in the roadside soil and dust around Kamikochi, Chubu Sangaku National Park, Japan. Geochemical Journal.

[CR37] Potgieter-Vermaak S, Horemans B, Anaf W, Cardell C, Van Grieken R (2012). Degradation potential of airborne particulate matter at the Alhambra monument: A Raman spectroscopic and electron probe X-ray microanalysis study. Raman Spectroscopy.

[CR38] Potgieter-Vermaak S, Rotondo G, Novakovic V, Rollins S (2012). Component-specific toxic concerns of the inhalable fraction of urban road dust. Environmental Geochemistry and Health.

[CR39] Rodrigues SM, Coehlo C, Cruz N, Monteiro RJR, Henriques B, Duarte AC, Romkens PFAM, Pereira E (2014). Oral bioaccessibility and human exposure to anthropogenic and geogenic mercury in urban, industrial and mining areas. Science of the Total Environment.

[CR40] Sondreal EA, Benson SA, Pavlish JH, Ralston NVC (2004). An overview of air quality III: Mercury, trace elements and particulate matter. Fuel Processing Technology.

[CR41] Sturm R (2013). Theoretical models for the simulation of particle deposition and tracheobronchial clearance in lungs of patients with chronic bronchitis. Annals of Translational Medicine.

[CR42] Sysalova J, Kucera J, Drtinova B, Cervenka R, Zverina O, Komarek J, Kamenik J (2017). Mercury species in formerly contaminated soils and released soil gases. Science of the Total Environment.

[CR43] Taylor K, Robertson D (2009). Electron microbeam analysis of urban road-deposited sediment, Manchester, UK: Improved source discrimination and metal speciation assessment. Applied Geochemistry.

[CR44] Tipping E, Lofts S, Hooper H, Frey B, Spurgeon D, Svedsen C (2010). Critical limits for Hg(II) in soils, derived from chronic toxicity data. Environmental Pollution.

[CR52] Tipping E, Poskitt JM, Lawlor AJ, Wadsworth RA, Norris DA, Hall JR (2011). Mercury in United Kingdom topsoils: Concentrations, pools, and critical limit exceedances. Environmental Pollution.

[CR50] USEPA (1996). SW-846 test method 3052: Microwave assisted acid digestion of siliceous and organically based matrices. https://www.epa.gov/hw-sw846/sw-846-test-method-3052-microwave-assisted-acid-digestion-siliceous-and-organically-based. Accessed 12 June 2019.

[CR45] Vane CH, Jones DG, Lister TR (2009). Mercury contamination in surface sediments and sediment cores of the Mersey Estuary, UK. Marine Pollution Bulletin.

[CR46] Walters C, Couto M, McClurg N, Silwana B, Somerset V (2017). Baseline monitoring of mercury levels in environmental matrices in the Limpopo Province. Water Soil and Air Pollution.

[CR47] Wang Z, Wang J, Kouketsu Y, Bodnar RJ, Gill BC, Xiao S (2017). Raman geothermometry of carbonaceous material in the basal Ediacaran Doushantuo cap dolostone: The thermal history of extremely negative δ^13^C signatures in the aftermath of the terminal Cryogenian snowball Earth glaciation. Precambrian Research.

[CR48] WHO. (1990). Environmental health criteria 101, methylmercury. http://www.inchem.org/documents/ehc/ehc/ehc101.htm. Last accessed April 30, 2018.

[CR49] Wragg J, Klinck B (2007). The bioaccessibility of lead from Welsh mine waste using a respiratory uptake test. Journal of Environmental Science and Health, Part A: Toxic/Hazardous Substances & Environmental Engineering.

