# Supplementary material for: A scoping study of component-specific toxicity of mercury in urban road dusts from three international locations
Source: Environ Geochem Health. 2019 Jun 18;42(4):1127–39. doi: 10.1007/s10653-019-00351-1 (PMC7225195; doi:10.1007/s10653-019-00351-1)
Supplement: Supplementary file 1 — Supplementary material 1 (DOCX 14 kb) [file 10653_2019_351_MOESM1_ESM.docx]

Supplementary Section – Mercury Clustering

Raman Spectroscopy can be used for gaining a comprehensive assessment of individual particles (Taylor and Robertson 2009; Potgieter-Vermaak *et al.* 2012a). These studies, concerned with characterising individual particles, show that there is scope for analysing particles within the interest of health. However, the process can only be considered qualitative due to number of particles analysed. Incorporating the process with a quantitative method, such as scanning electron microscope with energy dispersive X-ray microanalysis (SEM-EDX) has the potential to provide a more quantitative approach.

Kandler et al. (2011) have shown that particles can be clustered based on atomic ratios, determined by automated scanning electron microscope with energy dispersive X-ray microanalysis (SEM-EDX), to obtain a class of particle (silicates, calcites etc.). However, the analysis fails to inform us how these elements are present. Anaf et al. 2012 showed that clustering can be done based on atomic ratios. For example considering the chemical formula for dolomite, CaMg(CO_3_)_2_, where the atomic ratio of Ca : Mg is 1 : 1. This then informs one of the criterion within the clustering criteria for dolomite cited by Anaf et al. (2012), which is 0.33 < Mg/Ca < 3 and Mg and Ca denote the moles for each element, calculated from the elemental concentration obtained by EDX for that specific particle. This represents a large margin of error for the atomic ratio, however the other criterion used to define a dolomite particle is Mg+Ca/AE > 0.45, where AE represents the sum of moles of every other element quantified. This criterion is used to ensure that the main constituents of the particle are Mg and Ca, therefore with the albeit relaxed atomic ratio there is a large degree of certainty that this particle is dolomite.

As the aim of this study is to better understand the microchemical structure of individual particles containing mercury and that influences the toxicity, the methods used by Kandler et al. (2011) and Anaf et al. (2012) are adapted for use with mercury compounds. This study assumes that a large amount of mercury containing compounds are present as inclusions on larger particles, rather than existing as individual mercury rich particles. Therefore, it may be necessary to dispense of clustering criterion which characterise a compound as the main constituent of a particle and place more emphasis on the stoichiometric ratios between elements of known mercury compounds but in a stricter manner, to ensure their presence, once mercury containing particles have been identified qualitatively by Raman Spectroscopy.

Clustering the data will enable multi-elemental associations of mercury compounds. The presence of various mercury compounds were investigated by setting chemical boundaries according to their chemical composition and following the Kandler et al. (2011) approach. In that way the atomic ratio criteria used for classification of azarkite were set as 1.35<Hg/S<1.65 and 1.35<Br/S<1.65, for example. Using this approach for CC-SEM-EDX data, it was found that one azarkite particles was observed in the Manchester Samples, none in the Brazilian samples and none in the South Africa samples. Azarkite is a suitable mercury compound to set up criteria for in this analysis as the presence has been confirmed in previous Raman spectroscopy work on samples of this nature (Potgieter-Vermaak et al. 2012a). Another noteworthy conclusion was that only the Manchester samples contained mercuric chloride, which is fairly soluble in acidic solutions and could potentially explain why the Manchester samples showed the highest bioaccessibility. Other mercury compounds investigated are displayed in Table S1, It is noteworthy that the inclusion of these compounds is speculative and should be treated with due uncertainty as no mercury particles were observed qualitatively using Raman Spectroscopy. The purpose of including it within this supplementary section is to exhibit this approach as potentially useful with a scope to combining qualitative and quantitative techniques. The limitation here is the time-consuming nature of Raman Spectroscopy as a means for qualitative analysis. Quantities of each of these compounds observed are displayed in Table S2.

Table S1. Mercury clustering compounds, criteria used.

| **Compound** | **Criteria** |
| --- | --- |
| Azarkite (Hg_3_S_2_Br_2_) | 1.35<Hg/S<1.65 and 1.35<Br/S<1.65 |
| Cinnebar (HgS) | 0.9<Hg/S<1.1 |
| Danielsite (HgS_8_(CuAg)_14_) | 0.1125<Hg/S<0.1375 and 0.06429<Hg/Cu<0.07857 |
| HgCl_2_ | 0.3<Hg/Cl<0.7 |
| Edoylerite (Hg_3_(CrO_4_)S_2_) | 1.35<Hg/S<1.65 and 2.7<Hg/Cr<3.3 |
| Fettelite (As_4_Hg) | 0.225<Hg/As<0.275 |
| Galkite ((Hg_5_Cu)CsAs_4_S_12_) | 1.125<Hg/As<1.375 and 4.5<Hg/Cu<5.5 |

Table S2. Mercury Clustering observations for each sample

| **Sample** | **Azarkite** | **Cinnebar** | **Danielsite** | **HgCl_2_** | **Edoylerite** | **Fettelite** | **Galkite** |
| --- | --- | --- | --- | --- | --- | --- | --- |
| Man <38 µm | 1 | 1 | 0 | 126 | 0 | 1 | 0 |
| Man 63-38 µm | 0 | 0 | 0 |  | 0 | 0 | 0 |
| Man 125-63 µm | 0 | 2 | 0 |  | 0 | 0 | 0 |
| Joh <50 µm | 0 | 1 | 0 | 0 | 0 | 0 | 0 |
| Joh 100-50 µm | 0 | 0 | 0 | 0 | 0 | 0 | 0 |
| Cur <38 µm | 0 | 0 | 0 | 0 | 0 | 0 | 0 |
| Cur 63-38 µm | 0 | 0 | 0 | 0 | 0 | 0 | 0 |
| Cur 125-63 µm | 0 | 0 | 0 | 0 | 0 | 0 | 0 |

This analysis of the CC-SEM-EDX instrumentation fails to provide any robust or conclusive results, but it is the opinion of the authors that the application is worthy of further testing and may provide a useful insight into the form in which mercury is present in environmental samples. Further investigations into the materials which Hg is observed with using CC-DEM-EDX may assist in elucidating this, along with a more in-depth study into the Raman active Hg containing particles.
